# A prospective randomized study of arthroscopic ACL reconstruction with adjustable- versus fixed-loop device for femoral side fixation

**DOI:** 10.1186/s43019-021-00124-0

**Published:** 2021-12-04

**Authors:** Naiyer Asif, Mohammad Jesan Khan, K. P. Haris, Shah Waliullah, Anubhav Sharma, Danish Firoz

**Affiliations:** 1 Department of Orthopaedic Surgery, Faculty of Medicine, JNMCH, AMU, Aligarh, Uttar Pradesh India; 2 Department of Orthopaedics, KGMU, Lucknow, Uttar Pradesh India

**Keywords:** Anterior cruciate ligament, Adjustable-loop device, Fixed-loop device

## Abstract

**Purpose:**

Suspensory devices are extensively used in the management of anterior cruciate ligament (ACL) tear. They include fixed- and adjustable-loop devices. There are only a few studies comparing the efficacy of these two devices in the available literature. Therefore, the aim of this study is to compare clinical outcomes between the adjustable-loop device (group I) and fixed-loop device (group II).

**Materials and methods:**

This was a prospective randomized study. Both groups were equivalent in demographic, preoperative, and intraoperative variables. Twenty-three patients underwent femoral side graft fixation with adjustable-loop and 20 with fixed-loop devices. Four patients were lost to follow-up. Assessment of clinical outcome was done with International Knee Documentation Committee (IKDC) score, Lysholm score, and knee stability tests (Lachman test and pivot shift test). Patient evaluation was performed preoperatively and finally postoperatively 2 years after surgery.

**Results:**

Postoperative IKDC scores of group I and II were 91.9 ± 3.6 and 91.5 ± 3.6, respectively, and Lysholm scores were 91.0 ± 3.6 and 91.4 ± 3.5, respectively, after 2 years; however, the difference in the outcomes was statistically insignificant (*p* > 0.05). Twenty patients (87%) in group I and 17 patients (85%) in group II had a negative Lachman test (*p* = 0.8). Twenty-two patients (95.7%) in group I and 19 patients (95%) in group II had a negative pivot shift test (*p* = 0.9).

**Conclusion:**

ACL reconstruction with fixed- and adjustable-loop suspensory devices for graft fixation gives equivalent and satisfactory clinical results.

**Level of evidence:**

1.

## Introduction

Arthroscopic anatomic reconstruction is the preferred surgical option for anterior cruciate ligament (ACL) tears [1]. Normal knee kinematics and knee stability are restored with ACL reconstruction. Its foremost intent is to impart a strong graft fixation initially so that tendon-to-bone healing and graft integration can take place inside bony tunnels. The fixation should be able to provide enough strength to prevent slipping under the effect of repeated loading and progressive loosening during the initial postoperative phase. Common femoral graft fixation options comprise compression and suspensory devices [2]

Cortical suspensory fixation is considered an ideal model of femoral fixation. It is extensively used worldwide [3]. One such fixed-loop device is EndoButton (EB) CL (Smith & Nephew Inc., Andover, MA, USA). Additional drilling of the femoral tunnel is required so that the button comes out of the lateral femoral cortex. This leaves some part of the socket devoid of graft where graft motion can take place. This can further lead to the widening of the tunnel and jeopardize graft incorporation inside the tunnel [3]. Moreover, anatomical tunnel creation can sometimes result in short tunnel length and inadequate graft length inside the bone [4]. To address these shortcomings, second generation adjustable suspensory loop fixation devices were innovated TightRope (TR) (Arthrex Inc., Naples, FL, USA). These devices do not require over-drilling. Their loop can be tightened and adjusted according to the tunnel length during the surgical process, thereby decreasing the possibility of bungee cord effect [3, 5, 6].

However, recent biomechanical studies have shown a considerable amount of loosening in adjustable-loop devices, which may affect clinical results after ACL reconstruction [3, 4, 7–9]. Until now, only a few randomized studies have compared clinical results of these devices following reconstruction with hamstring graft [6, 10–12]. The available body of literature suggests a lack of sufficient evidence to recommend the use of fixed or adjustable suspensory fixation devices [13]. High-quality randomized studies are desired to formulate suitable guidelines regarding selecting the type of cortical suspension devices. Our study aimed to compare clinical results between fixed- and adjustable-loop devices in terms of knee scoring systems and laxity assessment. It was hypothesized that the clinical outcome of fixed-loop devices (FDL) would be better than adjustable-loop devices (ADL).

## Materials and methods

This was a prospective study of patients who presented to our hospital outpatient department/arthroscopy and Sports clinic from November 2016 to October 2018 with an ACL tear. Patients were diagnosed with ACL tear based on positive history of knee instability and knee laxity on clinical examination, supported by magnetic resonance imaging (MRI) evaluation.

Inclusion criteria consisted of patients between 18 and 50 years of age with ACL rupture or ACL rupture with meniscus injury presenting with knee instability. The exclusion criteria consisted of patients with severe osteoarthritic changes (Kellgren and Lawrence grades 3 and 4) [14] in the knee joint, any prior intra/extraarticular ligament surgery, patients with intra- or extraarticular ligament injury other than an ACL injury, and ACL injury associated with intraarticular fracture.

**Fig 1:**
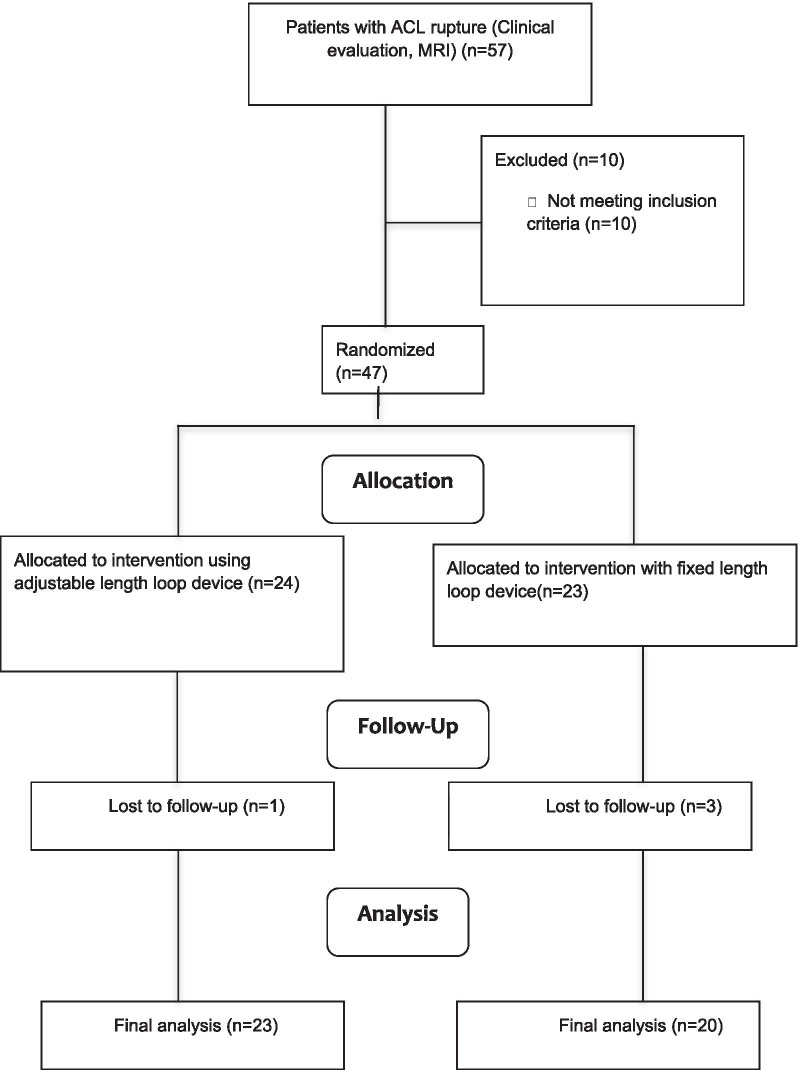
Flowchart showing randomization of patients into two groups

In our study, 47 patients with complete ACL tear were included for anatomical arthroscopic single-bundle reconstruction with quadrupled hamstring graft (semitendinosus and gracilis). Patients were then distributed to respective groups depending upon computer-based randomization (Fig. 1). In group I, adjustable-length loop device TR, and in group II fixed-length loop device EB was used for femoral side fixation and a bioabsorbable screw was used on the tibial side. Approval for the study was granted from the institutional ethics committee, and informed consent was taken from all patients. Four patients were lost to follow-up (one in group I and three in group II), hence 43 patients were available for the final analysis (23 in group I and 20 in group II). Demographic, preoperative, and intraoperative parameters were comparable between the two groups (Table 1).

**Table 1:** Demographic parameters and preoperative functional assessment

	Group I	Group II	*p*-Value
Gender			
Male	23	19	0.3^a^
Female	0	1	
Age (years)	26.7 ± 5.9 (18–40)	26.5 ± 8.3 (16–50)	0.9^b^
Time from injury to surgery (months)	8.5 ± 8.7 (2–36)	9.9 ± 15.2 (1–72)	0.7^b^
Side involved			0.6^a^
Right	12 (52.1%)	11 (55%)	
Left	11 (47.9%)	09 (45%)	
Medial meniscus			
Normal	15 (65.2%)	9 (45%)	0.2^a^
Tear	8 (34.7%)	11 (55%)	
Lateral meniscus			
Normal	18 (78.2%)	15 (75%)	0.8^a^
Tear	5 (21.8%)	5 (25%)	
Femoral tunnel length (mm)	39.2 ± 4.0 (33–45)	40.9 ± 3.2 (34–45)	0.1^b^
Graft diameter(mm)	8.4 ± 0.6 (8–10)	8.3 ± 0.6 (7–9)	0.5^b^
Quadrupled graft length (mm)	89.6 ± 8.2 (80–110)	93.5 ± 6.0 (80–100)	0.1^b^
Graft length in femoral tunnel (mm)	24.3 ± 1.7 (20–25)	24.5 ± 1.5 (20–25)	0.6^b^

Group I = adjustable-loop device, Group II = fixed-loop device; values expressed as mean with standard deviation in parentheses, range and percentages in brackets

^a^Fisher’s exact test, ^b^unpaired Student’s *t* test

A senior knee surgeon did all the surgeries, and a tourniquet was used in all cases. After confirming an ACL tear arthroscopically, ipsilateral hamstring tendons were harvested. Partial meniscectomy was done in all cases of meniscal tear, and meniscal repair was not done in any of the cases. A femoral tunnel was prepared after hyperflexing the knee through the anteromedial portal. Thereafter, the tibial tunnel was created with the help of tibial jig keeping an angle of 55°. The circumference of the femoral tunnel was guided by the thickness of the quadrupled hamstring graft. It was fixed on the femoral side either with EB or TR depending upon randomization. Additional drilling of the femoral tunnel by 10 mm more than the expected intraosseous graft length was done only in the EB group, while it was not required in the TR group. Interference screw (bioabsorbable screw) was used for fixation of graft in the tibial tunnel in both the groups [10, 15]. Recycling of the knee was done 20 times to get rid of any residual graft creep. Under the direct arthroscopic vision, the ACL was probed to assess laxity. Retensioning was done by pulling the alternating white strands until the ACL graft was fully taut and completely seated. Examination of ACL in flexion and extension was done to ensure graft tautness and femoral notch impingement [16].

On the second postoperative day, patients were allowed to bear weight as tolerated with the help of crutch/walker, with gradual flexion of knee started aiming to achieve 90° of flexion until 3 weeks, and were discharged on day three with home physiotherapy recommendation. Patients were given a knee immobilizer for 4 weeks, and then the brace was discontinued according to the patient’s comfort. Sports activity was performed after 6–8 months.

Functional results were evaluated by two independent observers who were blinded to the type of device used, through International Knee Documentation Committee (IKDC) scoring and Lysholm scoring [17]. Lachman tests and pivot shift tests were used to evaluate knee stability [18, 19]. Measurement of the quadriceps wasting was done at a point taken 15 cm proximal to the superior pole of patella [20]. The assessment was done 2 years after surgery.

SPSS software, version 20.0 (IBM Corp., Chicago) was utilized for the statistical assessment. The unpaired Student’s *t* test (b) and the paired *t* test (c) were applied for continuous data, while the Fisher’s exact test (a) was applied for categorical data. Statistical significance was considered for a *p* value  < 0.05. Sample size was calculated based on the IKDC score, the primary endpoint of this study [21, 22]. The follow-up IKDC score of groups I and II were postulated as 86.5 ± 6.5 and 77.5 ± 13, respectively, which was taken from a study done earlier by Tsoukas et al. [23] For a power of 0.8 and alpha value of 0.05, the calculated sample size was 22. To retain power of 80%, we took the same sample in both fixed- and adjustable-loop groups.

## Results

Demographic data and preoperative functional scores of the study patients were analyzed (Table 1). Both groups were analogous with respect to preoperative scores.

All patients were clinically examined preoperatively through the Lachman test and pivot shift tests (Table 2). In group I, 6 patients (26%) had positive Lachman grade 2 and 16 patients (69.7%) had positive grade 3, whereas in group II, 5 patients (25%) had grade 2 and 14 patients (70%) had grade 3 instability (*p* = 0.9). Pivot shift test was grade 2 in 13 patients (56.5%) and grade 3 in 8 patients (34.8%) in group I; however, in group II it was grade 2 in 11 patients (55%) and grade 3 in 7 (35%) patients (*p* = 0.9). Preoperative IKDC scores of group I and group II were 56.3 ± 8.1 (40.2–66.7) and 52.6 ± 8.7 (40.2–66.7) (*p* = 0.2), respectively, and Lysholm scores in groups I and II were 54.6 ± 4.8 (44–67) and 56.1 ± 3.9 (48–62), respectively (*p* = 0.3). The average wasting of quadriceps was 1.7 ± 0.9 cm in group I and 1.8 ± 0.9 cm in group II (*p* = 1.0).

**Table 2:** Preoperative clinical assessment

	Group I	Group II	*p*-Value
Lachman test			
Grade 0	0 (0%)	0 (0%)	0.9^a^
Grade 1	1 (4.3%)	1 (5%)	
Grade 2	6 (26%)	5 (25%)	
Grade 3	16 (69.7%)	14 (70%)	
Pivot shift test			
Grade 0	0 (0%)	0 (0%)	0.9^a^
Grade 1	2 (8.7%)	2 (10%)	
Grade 2	13 (56.5%)	11 (55%)	
Grade 3	8 (34.8)	7 (35%)	
IKDC score	56.3 ± 8.1 (40.2–66.7)	52.6 ± 8.7 (40.2–66.7)	0.2^b^
Lysholm score	54.6 ± 4.8 (44–67)	56.1 ± 3.9 (48–62)	0.3^b^
Index thigh atrophy (cm)	1.7 ± 0.9 (01–04)	1.8 ± 0.9 (01–04)	1.0^b^

Group I = adjustable-loop device, Group II = fixed-loop device, IKDC = International Knee Documentation Committee, values are expressed as mean with standard deviation in parenthesis, range, and percentages in brackets

Final clinical and functional assessments of all patients were done 2 years after the surgery. There were three patients in each group who had positive Lachman grade 1 (*p* = 0.8); however, pivot shift grade 1 was present in one patient in each group (*p* = 0.9) (Table 3). Postoperative (Table 4) Lysholm scores in groups I and II were 91.0 ± 3.6 (82–95) and 91.4 ± 3.5 (82–96), respectively (*p* < 0.001), and postoperative IKDC scores in groups I and II were 91.9 ± 3.6 (86.2–97.7) and 91.5 ± 3.6 (87.4–96.6), respectively (*p* < 0.001). The average wasting of quadriceps was 1.0 ± 0.6 cm in group I and 1.1 ± 0.6 cm in group II (Table 4) (*p* < 0.001).

**Table 3:** Postoperative clinical assessment at 2 year

	Group I	Group II	*p*-Value
Lachman test			
Grade 0	20 (87%)	17 (85%)	0.8^a^
Grade 1	3 (13%)	3 (15%)	
Grade 2	0 (0%)	0 (0%)	
Grade 3	0 (0%)	0 (0%)	
Pivot shift test			
Grade 0	22 (95.7%)	19 (95%)	0.9^a^
Grade 1	1 (4.7%)	1 (5%)	
Grade 2	0 (0%)	0 (0%)	
Grade 3	0 (0%)	0 (0%)	
Change in IKDC score	35.7 ± 9.0	38.9 ± 8.8	0.3^b^
Change in Lysholm score	36.4 ± 5.5	35.3 ± 5.2	0.5^b^
Change in thigh circumference wasting (cm)	0.7 ± 0.8	0.7 ± 0.8	0.9^b^

Group I = adjustable-loop device, Group II = fixed-loop device, IKDC = International Knee Documentation Committee, values expressed as mean with standard deviation in parentheses, range, and percentages in bracket

**Table 4:** Functional assessment in both groups

	Group I			Group II		
	Preoperative	Postoperative	*p*-Value	Preoperative	Postoperative	*p*-Value
IKDC score	56.3 ± 8.1 (40.2–66.7)	91.9 ± 3.6 (86.2–97.7)	< 0.001^C^	52.6 ± 8.7	91.5 ± 3.6 (87.4–96.6)	< 0.001^C^
				(40.2–66.7)		
Lysholm score	54.6 ± 4.8 (44–67)	91.0 ± 3.6 (82–95)	< 0.001^C^	56.1 ± 3.9	91.4 ± 3.5 (82–96)	< 0.001^C^
				(48–62)		
Thigh circumference wasting (cm)	1.7 ± 0.9 (01–04)	1.0 ± 0.6 (0–2.5)	< 0.001^C^	1.8 ± 0.9 (01–04)	1.1 ± 0.6 (0–02)	< 0.001^C^

Group I = adjustable-loop device, Group II = fixed-loop device, values are expressed as mean, with standard deviation in parentheses, range, and percentages in brackets, C = paired *t*-test

The mean IKDC score, Lysholm score, and thigh circumference increased significantly after surgery (*p* < 0.05) in their respective groups (Table 4). However, on comparing the changes in IKDC score (*p* = 0.3), Lysholm score (*p* = 0.5), and thigh circumference (*p* = 0.9) between the two groups, the difference was not statistically significant (Table 3). Superficial infection was seen in three patients (one patient in group I and two patients in group II) and were treated conservatively. There was no graft failure, and the range of motion was full (0–140°) in both groups.

## Discussion

Most of the patients were initially in instability grades 2 and 3, as assessed by pivot shift and Lachman tests, which indicated reconstruction. Knee stability was restored in most patients as 87% in group I and 85% in group II tested negative on the Lachman test. Similarly, a negative pivot shift test was observed in 95.7% of group I and 95% of group II. Ahn et al. [11] reported 72.7% negative Lachman tests in the fixed-loop device group and 88.2% in the adjustable-loop device group. Furthermore, they found 81.8% negative pivot shift test in the fixed-loop device group and 88.2% in the adjustable-loop device group. Choi et al. [6] found a negative Lachman test in 70.1% of the fixed-loop device group and 82% in the adjustable-loop device group, and a negative pivot shift test in 74.6% of the fixed-loop device group and 80% in the adjustable-loop device group. Boyle et al. [24] did not find any patient with a positive Lachman test or pivot shift test in either group. Our results were comparable to the studies mentioned above, probably because all the authors used hamstring autograft and similar types of cortical suspension devices.

Significant improvement was noted in functional outcomes in the form of Lysholm and IKDC scores in both groups (Table 4). Sheth et al. [13] found Lysholm scores of 94.23 and 94.32 in fixed and adjustable groups, respectively. They also found an IKDC score of 92.03 in the fixed and 92.16 in the adjustable group. Ranjan et al. [10] reported an equal Lysholm score of 91.8 in both the groups, whereas they had found the IKDC score as being 85.2 in the fixed and 84.3 in the adjustable group. Ahn et al. [11] identified IKDC scores of 79.43 in the fixed-loop device and 78.6 in the adjustable group. Choi et al. [6] found Lysholm scores of 92.6 in the fixed-loop device and 94.3 in the adjustable-loop device group. Our observations matched with the studies mentioned above, as all our patients followed institutional physiotherapy protocol.

Short femoral tunnel length is a critical issue of the Anteromedial (AM) portal technique, especially when using a fixed-loop device. In a cadaveric study on a Western population, the average femoral tunnel length using the anteromedial portal method was 30.5 mm [25]. Furthermore, five subjects had less than 30 mm femoral tunnel length. In these cases, fixed-loop device use can be compromised because of the short graft length within the femoral tunnel. Many alternative techniques have been suggested to overcome this issue and secure adequate femoral length during the operation. Some authors reported that knee flexion with an anteromedial portal technique can yield a longer femoral tunnel; therefore, the knee joint's hyperflexion during the femoral tunnel drilling could avoid a short femoral socket [26, 27]. Other investigators proposed using a curved femoral guide and a flexible reamer that assists in making the adequate femoral tunnel length without hyperflexion [28, 29]. Since we created a femoral tunnel by drilling carefully with hyperflexion of the knee, we did not encounter any patient with a femoral tunnel less than 30 mm.

Some in vitro studies indicate that adjustable devices are biomechanically inferior to fixed devices. In one biomechanical analysis, the authors found increased displacement of the adjustable loop due to slippage of the device when tested under cyclic and pull to failure loading [8]. In another biomechanical study, the authors noticed a momentous lengthening in a loop (> 3 mm) during cyclic testing because of suture [4]. Other investigators also found similar results in their biomechanical studies [7, 9]. One researcher observed a significant difference between the two devices during the displacement testing of individual devices; however, testing the construct with porcine bone and bovine tendon did not show any significant difference in displacement between the two devices [3]. Despite these facts, some authors could not appreciate any noteworthy difference between the two devices regarding knee stability and graft failure [10, 24]. Other investigators did not observe any notable difference in tunnel widening and clinical results between the two devices using hamstring grafts [6, 12]. In our study, functional results and knee laxity assessment at last follow-up were statistically not different in either the adjustable- or fixed-loop groups. Our findings correlate with many in vivo studies; however, it is one of the few randomized studies conducted in this context.

Biomechanical studies and their findings usually do not match with clinical studies. The laboratory studies can never provide a situation that can accurately simulate biomechanical and physiological loads of the adjustable loop at the femoral fixation site. Moreover, in vitro specimens are built to replicate physiologic loads, which is seldom applicable in a clinical scenario. Furthermore, the laboratory-based studies can never recreate the complicated forces subjected to an adjustable device in vivo [10].

There are a few limitations to the study. Firstly, knee stability was assessed by subjective methods, and an arthrometer was not used. Secondly, tunnel widening was also not measured. Thirdly, there are minor differences between FDL and ADL in terms of surgical techniques, which can only be established with more objective data. Finally, the sample size was limited, and prolonged follow-up could not be done.

## Conclusions

The authors did not find a significant clinical difference between the two groups. Fixed- and adjustable-loop suspensory devices are reasonably efficient for fixing femoral side grafts in arthroscopic ACL reconstruction, and give equivalent outcomes. This study confirmed that lengthening associated with adjustable-loop devices in biomechanical studies may not be relevant in clinical settings. More randomized studies with a larger sample size and longer follow-up are warranted to corroborate our findings.

## Data Availability

All data generated or analyzed during this study are included in this published article [and its supplementary information files, especially in Tables].

## References

[1] Fu FH, Bennett CH, Ma CB, Menetrey J, Lattermann C (2000). Current trends in anterior cruciate ligament reconstruction. Part II. Operative procedures and clinical correlations. Am J Sports Med.

[2] Zeng C, Lei G, Gao S, Luo W (2013). Methods and devices for graft fixation in anterior cruciate ligament reconstruction. Cochrane Database Syst Rev.

[3] Eguchi A, Ochi M, Adachi N, Deie M, Nakamae A, Usman MA (2014). Mechanical properties of suspensory fixation devices for anterior cruciate ligament reconstruction: comparison of the fixed-length loop device versus the adjustable-length loop device. Knee.

[4] Barrow AE, Pilia M, Guda T, Kadrmas WR, Burns TC (2014). Femoral suspension devices for anterior cruciate ligament reconstruction: do adjustable loops lengthen?. Am J Sports Med.

[5] Lubowitz JH, Ahmad CS, Amhad CH, Anderson K (2011). All-inside anterior cruciate ligament graft-link technique: second-generation, no-incision anterior cruciate ligament reconstruction. Arthrosc J Arthrosc Relat Surg.

[6] Choi N-H, Yang B-S, Victoroff BN (2017). Clinical and radiological outcomes after hamstring anterior cruciate ligament reconstructions: comparison between fixed-loop and adjustable-loop cortical suspension devices. Am J Sports Med.

[7] Johnson JS, Smith SD, LaPrade CM, Turnbull TL, LaPrade RF, Wijdicks CA (2015). A biomechanical comparison of femoral cortical suspension devices for soft tissue anterior cruciate ligament reconstruction under high loads. Am J Sports Med.

[8] Petre BM, Smith SD, Jansson KS, de Meijer P-P, Hackett TR, LaPrade RF (2013). Femoral cortical suspension devices for soft tissue anterior cruciate ligament reconstruction: a comparative biomechanical study. Am J Sports Med.

[9] Noonan BC, Dines JS, Allen AA, Altchek DW, Bedi A (2016). Biomechanical evaluation of an adjustable loop suspensory anterior cruciate ligament reconstruction fixation device: the value of retensioning and knot tying. Arthrosc J Arthrosc Relat Surg.

[10] Ranjan R, Gaba S, Goel L, Asif N, Kalra M, Kumar R (2018). In vivo comparison of a fixed loop (EndoButton CL) with an adjustable loop (TightRope RT) device for femoral fixation of the graft in ACL reconstruction: a prospective randomized study and a literature review. J Orthop Surg.

[11] Ahn JH, Ko TS, Lee YS, Jeong HJ, Park JK (2018). Magnetic resonance imaging and clinical results of outside-in anterior cruciate ligament reconstruction: a comparison of fixed- and adjustable-length loop cortical fixation. Clin Orthop Surg.

[12] Lanzetti RM, Monaco E, Carli AD, Grasso A, Ciompi A, Sigillo R (2016). Can an adjustable-loop length suspensory fixation device reduce femoral tunnel enlargement in anterior cruciate ligament reconstruction? A prospective computer tomography study. Knee.

[13] Sheth H, Salunke AA, Barve R, Nirkhe R (2019). Arthroscopic ACL reconstruction using fixed suspensory device versus adjustable suspensory device for femoral side graft fixation: what are the outcomes?. J Clin Orthop Trauma.

[14] Kohn MD, Sassoon AA, Fernando ND (2016). Classifications in brief: Kellgren-Lawrence classification of osteoarthritis. Clin Orthop.

[15] Sundararajan SR, Sambandam B, Singh A, Rajagopalakrishnan R, Rajasekaran S (2018). Does second-generation suspensory implant negate tunnel widening of first-generation implant following anterior cruciate ligament reconstruction?. Knee Surg Relat Res.

[16] Gamboa JT, Shin EC, Pathare NP, McGahan PJ, Chen JL (2018). Graft retensioning technique using an adjustable-loop fixation device in arthroscopic anterior cruciate ligament reconstruction. Arthrosc Tech.

[17] Collins N, Misra D, Felson D, Crossley KM, Roos EM (2011). Measures of knee function. Arthritis Care Res.

[18] Mulligan EP, McGuffie DQ, Coyner K, Khazzam M (2015). The reliability and diagnostic accuracy of assessing the translation endpoint during the Lachman test. Int J Sports Phys Ther.

[19] Seo SS, Kim CW, Kim JG, Jin SY (2013). Clinical results comparing transtibial technique and outside in technique in single bundle anterior cruciate ligament reconstruction. Knee Surg Relat Res.

[20] Asif N, Ranjan R, Ahmed S, Sabir AB, Jilani LZ, Qureshi OA (2016). Prediction of quadruple hamstring graft diameter for anterior cruciate ligament reconstruction by anthropometric measurements. Indian J Orthop.

[21] Pocock S (1998). Clinical trials. A practical approach.

[22] Julious SA (2004). Sample sizes for clinical trials with normal data. Stat Med.

[23] Tsoukas D, Fotopoulos V, Basdekis G, Makridis KG (2016). No difference in osteoarthritis after surgical and non-surgical treatment of ACL-injured knees after 10 years. Knee Surg Sports Traumatol Arthrosc.

[24] Boyle MJ, Vovos TJ, Walker CG, Stabile KJ, Roth JM, Garrett WE (2015). Does adjustable-loop femoral cortical suspension loosen after anterior cruciate ligament reconstruction? A retrospective comparative study. Knee.

[25] Lubowitz J, Konicek J (2010). Anterior cruciate ligament femoral tunnel length: cadaveric analysis comparing anteromedial portal versus outside-in technique. Arthrosc J Arthrosc Relat Surg.

[26] Iyyampillai G, Raman ET, Rajan DV, Krishnamoorthy A, Sahanand S (2013). Determinants of femoral tunnel length in anterior cruciate ligament reconstruction: CT analysis of the influence of tunnel orientation on the length. Knee Surg Relat Res.

[27] Kim S-H, Kim S-J, Choi CH, Kim D, Jung M (2018). Optimal condition to create femoral tunnel considering combined influence of knee flexion and transverse drill angle in anatomical single-bundle ACL reconstruction using medial portal technique: 3D simulation study. BioMed Res Int.

[28] Kadija M, Milovanović D, Bumbaširević M, Carević Z, Dubljanin-Raspopović E, Stijak L (2015). Length of the femoral tunnel in anatomic ACL reconstruction comparison of three techniques. Knee Surg Sports Traumatol Arthrosc.

[29] Wein F, Osemont B, Goetzmann T, Jacquot A, Valluy J, Saffarini M (2019). Anteversion and length of the femoral tunnel in ACL reconstruction: in-vivo comparison between rigid and flexible instrumentation. J Exp Orthop.

